# Mutations of BRCA2 in canine mammary tumors and their targeting potential in clinical therapy

**DOI:** 10.1186/s12917-020-2247-4

**Published:** 2020-01-31

**Authors:** Pauline Thumser-Henner, Katarzyna J. Nytko, Carla Rohrer Bley

**Affiliations:** Division of Radiation Oncology, Vetsuisse Faculty, University of Zurich, Zurich, Switzerland

**Keywords:** Canine mammary cancer, BRCA2, PARP inhibitors, RAD51

## Abstract

Dogs develop cancer spontaneously with age, with breed-specific risk underlying differences in genetics. Mammary tumors are reported as the most frequent neoplasia in intact female dogs. Their high prevalence in certain breeds suggests a genetic component, as it is the case in human familial breast cancer, distinctly in BRCA2-associated cancers. However, the molecular genetics of BRCA2 in the pathogenesis of canine cancer are still under investigation.

Genetic variations of canine BRCA2 comprised single nucleotide polymorphisms, insertions and deletions. The BRCA2 level has been shown to be reduced in tumor gland samples, suggesting that low expression of BRCA2 is contributing to mammary tumor development in dogs. Additionally, specific variations of the BRCA2 gene affect RAD51 binding strength, critically damage the BRCA2-RAD51 binding and further provoke a defective repair. In humans, preclinical and clinical data revealed a synthetic lethality interaction between BRCA2 mutations and PARP inhibition. PARP inhibitors are successfully used to increase chemo- and radiotherapy sensitivity, although they are also associated with numerous side effects and acquired resistance. Cancer treatment of canine patients could benefit from increased chemo- and radiosensitivity, as their cancer therapy protocols usually include only low doses of drugs or radiation. Early investigations show tolerability of iniparib in dogs. PARP inhibitors also imply higher therapy costs and consequently are less likely to be accepted by pet owners.

We summarized the current evidence of canine BRCA2 gene alterations and their association with mammary tumors. Mutations in the canine BRCA2 gene have the potential to be exploited in clinical therapy through the usage of PARP inhibitors. However, further investigations are needed before introducing PARP inhibitors in veterinary clinical practice.

## Background

Cancer is the most common cause of death in dogs worldwide. For instance, it affects about 4 million dogs per year in the USA [1, 2]. A diverse range of cancers are observed in dogs. Age, nutrition, sex, reproductive status and environmental exposures are factors that influence canine tumor initiation and progression [3].

Mammary tumors are the most frequent type of tumor found in intact female dogs [4–6]. Certain breeds show high susceptibility to canine mammary cancer, indicative of an inheritable component [5, 7, 8]. Commonly, the dog’s owners notice tumors when macroscopic changes are already visible, or are found during a routine physical exam [9]. So far, surgical excision is the only effective treatment, consisting of the removal of altered glands and local lymph nodes. However, because of the high rate of metastases, surgery alone does not cure all canine patients [6, 10]. Consequently, in some cases chemotherapy or radiotherapy are used as adjuvant therapies [11]. Unfortunately, many tumor cells are showing resistances to theses therapeutics [12–14]. Thus, treatment of mammary tumors in dogs would benefit from additional therapies in order to increase the efficacy of chemo- and radiotherapy.

**Fig. 1:**
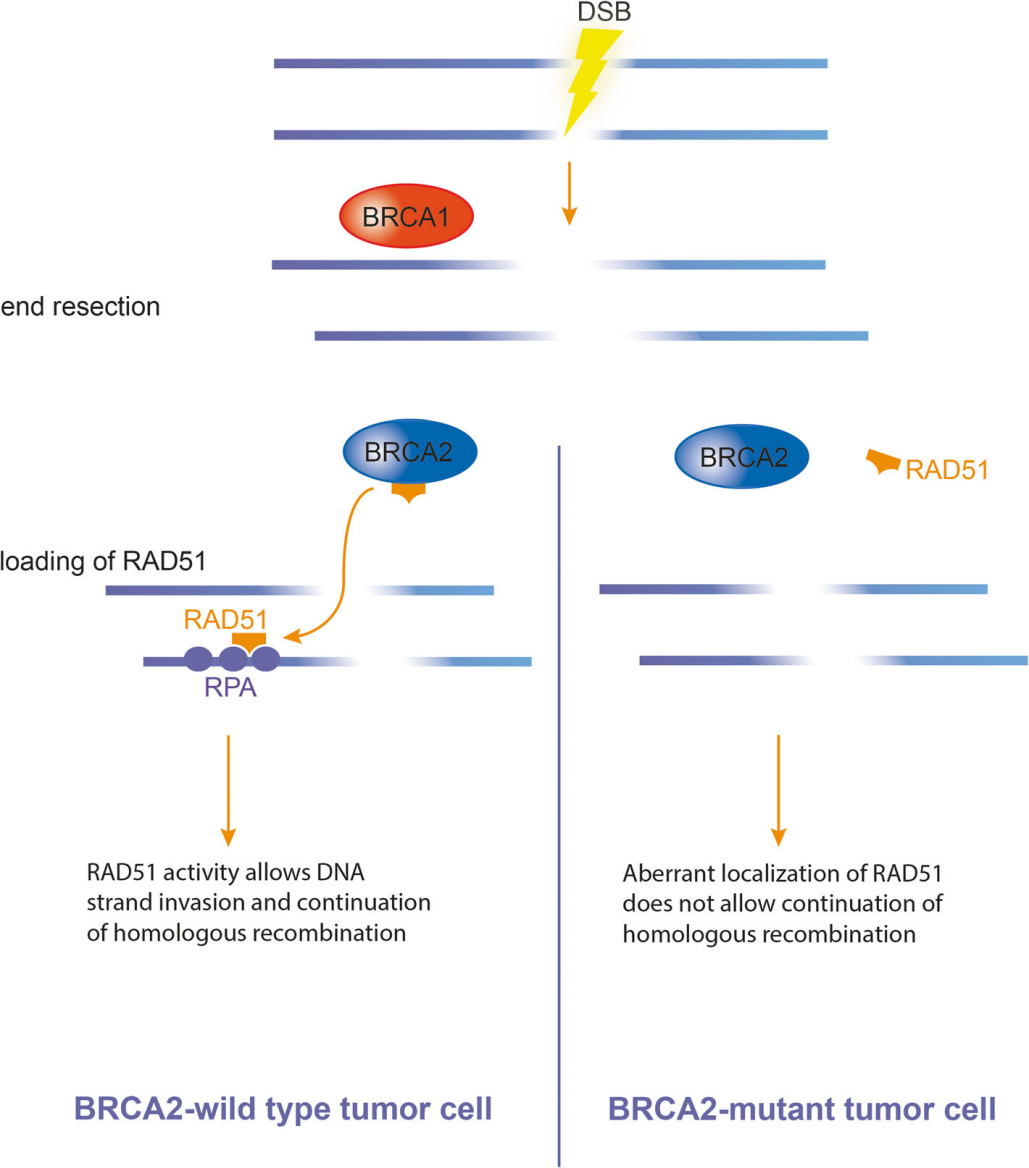
Simplified steps leading to BRCA2-RAD51 interaction after a break, depending on the BRCA2 mutation status. After formation of the DSB, DNA resection is dependent on BRCA1. BRCA2 then localizes RAD51 to the DNA, and RAD51 is loaded onto RPA-coated DNA to invade the DNA double helix. When the BRCA2 gene is mutated, and therefore the BRCA2 protein deficient, RAD51 cannot be efficiently localized onto DNA (figure based on: Wooster R. et al., 1995; Prakash R. et al., 2015; Shailani A. et al., 2018 [25, 27, 28])

Additionally, canine patients present genetic alterations that drive cancers, evidenced by the elucidation of the canine genome [15]. Example of these include alterations of p53 in canine mammary cancer and various cancer types as lymphoma and leukemia [16, 17], and mutations found in the tyrosine kinase growth factor receptor KIT in mast cell tumors of dogs [18, 19]. Thus, certain biomarkers of canine mammary tumors have been discovered and investigated in order to improve early detection of the tumors [20]. Among other gene mutations, mutations in the BRCA1/2 genes (Breast Cancer 1 and 2; their protein products are commonly called breast cancer type 1 or 2 susceptibility protein) have been reportedly associated with the development of mammary tumors in dogs [21–23]. Apart from being useful as biomarkers, BRCA1/2 have been also investigated as potential treatment targets [24]. Indeed, the wild-type BRCA2 gene is known as a tumor suppressor gene; BRCA2 maintains genome stability by its involvement in the repair of DNA double-strand breaks (DSBs) during homologous recombination [25, 26]. Homologous recombination occurs in the late S/G2 phase of the cell cycle and provides high-fidelity repair of DNA DSBs by using a sister chromatid or chromosome as a template. During the repair process, BRCA2 is attracted by BRCA1 to the place of damage and facilitates the loading of RAD51 protein onto RPA-coated (Replication Protein A) single-strand DNA, leading to RPA-RAD51 exchange (see Fig. 1). BRCA2 binds to RAD51 and localizes it to the nucleus, which is the site of DNA damage [25, 27, 28]. In BRCA2-mutated (deficient) cells, RAD51 is not transported into the nucleus and remains aberrantly in the cell. Ochiai et al. confirmed that canine BRCA2 protein also interacts with canine RAD51 [29, 30]. Hence, together with BRCA1, BRCA2 acts as a tumor suppressor; mutations in these genes will impede the cell’s ability to repair DNA damage, especially DNA DSBs. Damage can then accumulate in the cells, creating new mutations, pushing the cells towards becoming more prone to neoplastic transformation [27, 28, 31].

Thus, in human patients, women who present BRCA1 and/or BRCA2 mutations have a very high lifetime risk of developing breast and ovarian cancer [31]. Testing for BRCA1 and 2 mutations is nowadays used to screen for cancer susceptibility in women with a family history of breast or ovarian cancer [32]. Because BRCA2-deficient tumors present these particular type of defects, it has been exploited therapeutically through the principle of synthetic lethality. Synthetic lethality implies that the deficiency in the expression of one gene leaves the cell viable, but the perturbation of two genes simultaneously results in the loss of viability. Therefore, patients carrying germline mutations of BRCA2 are sensitive to a class of drugs called inhibitors of PARP (Poly(ADP-ribose) protein), because they have a specific type of DNA repair defect through the BRCA2 mutation [24]. The specific mutation status of these cells represents a diagnostic and therapeutic target that on the one hand explains the consequences of BRCA2 deficiency but on the other hand can be used to therapeutically attack the tumor cells [24, 33]. PARP inhibitors (or PARPi) have been clinically approved to exploit this principle and are now included in patients’ treatment, particularly in cases of BRCA2-mutated tumors. Patients carrying these germline mutations are responding to the drugs, because PARPi, such as olaparib, trap the single-strand breaks (SSBs) protein PARP1. Without specific SSB repair, the homologous recombination pathway is triggered, but in the absence of a functional BRCA2 protein, breaks accumulate, leading to more cell death [24, 33].

The present review aims to investigate the current knowledge about BRCA2 mutations in canine cancer and the consequence of identified polymorphisms on the interaction with RAD51 protein and discuss the potential of applying synthetic lethality in the treatment of canine mammary tumors.

## Main text

### Mechanisms for reduced BRCA2 expression in dogs

Rivera et al. showed that canine BRCA2 is associated with benign and malignant mammary tumors [22]. Out of 10 other human breast cancer genes, BRCA2 (and BRCA1) stood out as contributing to the risk of canine mammary tumors in the English springer spaniel [22].

Thus, Yoshikawa et al. later investigated the mRNA levels of BRCA2 in canine mammary tumor samples compared to mammary gland samples and found a significantly reduced level in the tumor samples, suggesting that low expression of BRCA2 contributes to mammary tumor development in dogs [23]. In contrast, a study conducted by Ripoli et al. did not show a significant difference in BRCA2 gene expression levels in between canine healthy tissue, malignant and benign tumors from fresh frozen samples [34]. However, in cases where a lower expression of BRCA2 is observed, what are the mechanisms triggering the tumor development?

BRCA2 mRNA levels seem to be likely reduced as result of a mutation occurring in the promoter region of the BRCA2 gene, as it was suggested by previous research using human samples [35, 36]. For instance, Maia et al. identified haplotypes having an impact on the expression profile of breast cells [35]. They showed several variants involved in altering the binding of transcription factors and mapped them to the promoter and two intronic regulatory elements of BRCA2. Thus, single nucleotide polymorphisms (SNPs) in the vincinity of the human BRCA2 gene seem to disturb the expression levels of BRCA2 mRNA and increase the breast cancer risk. Another mechanism that may reduce human BRCA2 expression is nonsense-mediated mRNA decay (NMD), a post-transcriptional quality control mechanism that ensures transcriptome fidelity by eliminating mRNAs containing premature termination codons (PTCs), avoiding the synthesis of truncated proteins [37]. Ware et al. found that in the human BRCA2 gene, some PTC-induced mutations, following alternative splicing, were associated with NMD [38]. Yoshikawa et al. investigated possible reasons for this reduction in expression in canine BRCA2 mRNA levels [23]. After identification and sequencing of the BRCA2 promoter region, the authors first highlighted that activity of the canine and human BRCA2 promoters were comparable, though some cis-elements in human BRCA2 promoter were not conserved. Additionally, variations located near the corresponding site of a human BRCA2 cis-element were found (nine allele types identified), but none of these alleles seemed to alter the canine BRCA2 promoter activity. Second, they examined whether the NMD system induced by PTCs was triggering BRCA2 level reductions. They detected two types of splice variants (they form the PTCs) in eight canine mammary tumor samples. One of the variant induced a frame-shift mutation and PTC that could lead to NMD [23].

In conclusion, a possible mechanism for reduced BRCA2 mRNA levels in canine tumors is nonsense-mediated mRNA decay. However, it is not yet completely clear whether mutations in the BRCA2 promoter region are involved.

### Polymorphisms found in the canine BRCA2 gene

The first finding of a polymorphic marker for canine BRCA2 was made by Yoshikawa et al. in 2005 (all following polymorphisms are summarized in Table 1) [39]. In their study, they found a single insertion/deletion polymorphism in the nuclear localization signal 2 (NLS2) of canine BRCA2, named 10204ins/delAAA. These nuclear localization signals (NLS1, 2 and 3) were shown in humans to have a role in the mislocalization of BRCA2 and thus in tumorigenesis in humans, as BRCA2 protein needs to be translocated from the cytoplasm to the nucleus to act in DNA damage repair [44, 45]. They showed that the NLS2 variation 10204insAAA causes an amino acid change, which enhances nuclear localization [39]. Their study further suggests that the translocation efficiency of BRCA2 may be associated with mammary tumor morbidity in dogs, because the morbidity rate of dogs was higher with AAA insertion than with AAA deletion [39].

**Table 1:** Variations found in the canine BRCA2 gene

Type of variation	Name/designation	Localization	Studied material	Reference
1 insertion/deletion	10204ins/delAAA	NLS2 (exon 27)	Blood samples of tumor-free dogs	[39]
2 missenses	10398A > G (Y3397C) 10421A > C (T3405P)	NLS3 (exon 27)	Blood/tissue samples of mammary tumor-bearing and tumor-free dogs	[40]
4 missenses	H143R, C386W, E643K, K1435R	histone acetyltransferase (HAT), P300/CBP-associated factor (P/CAF), BRC3	Tissue samples of canine mammary tumor	[41]
1 insertion	10,204 insertion AAA	NLS3	Tissue samples of canine mammary tumor	[41]
1 silence	7138C- > T	Fanconi anemia group G protein (FANCG)	Tissue samples of canine mammary tumor	[41]
1 SNP	ss748770619	Exon 24	Blood samples of canine mammary tumors	[21]
1 SNP	ss748770620	Exon 27	Blood samples of canine mammary tumors	[21]
1 SNP	2414 G > A	Exon 11	Tissue samples of canine mammary tumors	[42]
2 missenses	2414 A > G 2383 A > C	BRC4 (exon 11)	Tissue samples of canine mammary tumors	[42]
2 missenses	T1425P K1435R	BRC3	In silico analysis derived from mammary tumors	[43]

In a subsequent study made to investigate a suitable polymorphic marker for loss of heterozygosity, Yoshikawa et al. reported other polymorphisms [40]. Considering the genomic sequences of the exon 27 regions of mammary-tumor bearing and tumor-free dogs, they found four novel SNPs in addition to 10204ins/delAAA, some of them closely located to NLS3 [40].

The authors further concluded that this marker is not sufficient for an adequate loss of heterozygosity analysis of BRCA2. They investigated the establishment of novel polymorphic markers: from canine mammary tumors, they were able to highlight three novel genetic markers with high heterozygosity rates [41]. Because the heterozygosity rates were greater than 50%, they were sufficient to analyze the loss of heterozygosity. Additionally, they also report the first loss of heterozygosity in canine BRCA2, identified from a canine mammary tumor. In this dog, four novel missensse variations, one insertion variation and one silent variation were found, causing probably detrimental mutations.

Overall, genetic variations of canine BRCA2 comprised SNPs, insertions and deletions. However, both coding and non-coding SNPs have perhaps a role in tumorigenesis, affecting either protein function or transcription. By comparing sequences from mammary tumors and healthy mammary glands, Enginler et al. showed the existence of two SNPs in exon 24 and exon 27 of BRCA2, showing a significant association of exon 24 polymorphism in canine mammary tumors [21].

In addition, Hsu et al. investigated variations of exon 11 in malignant mammary tumors [42]. Exon 11, in both humans and dogs, is the largest exon and encodes the BRC repeats domains, conserved motifs that are crucial for interaction with Rad51 [46]. They identified multiple variations among 11 canine mammary tumors. These SNPs were for the most part missense mutations that could elicit structural changes in BRCA2 protein and silent mutations that do not provoke amino acid alteration [42] but may influence protein folding, as it was previously proposed [47, 48].

Maués et al. investigated canine BRCA2 exon 11 as well, evaluating the frequency of exon 11 SNPs in bitches with mammary tumors [49]. In their study, 97.9% of the bitches were affected by one to three SNPs in BRCA2 exon 11, suggesting a correlation between these gene polymorphisms and carcinogenesis [49].

Furthermore, Yoshikawa et al. investigated polymorphisms in the BRC repeats region of the BRCA2 gene [43]. They showed the T1425P and K1435R mutations in BRC3 in canine mammary tumor samples. As mentioned, the BRC repeats region has an important role in the interaction with the DNA repair protein RAD51, which is why mutations in this regions may imply a further effect on DNA repair, as we will address later on.

### How do these polymorphisms affect the interaction with RAD51?

The BRCA2 protein plays a key role in genome stability by recombining DNA and repair of DNA DSBs. BRCA2 interacts with the RAD51 protein, which catalyzes homologous DNA pairing and DNA strand exchange, and overexpression of BRCA2 and RAD51 is associated with poor prognosis in human cancer. The Rad51 gene has been identified also in dogs [50–52]. Ochiai et al. showed that canine BRCA2 protein interacts with canine RAD51 through the BRC repeats (BRC1–8, located in exon 11) and the extreme C-terminus [29, 30].

In addition, they later analyzed polymorphisms in canine BRC3 and in dogs of multiple breeds: they showed significant reduction of binding strength of BRC3–4 containing the a specific allele version (1425P) with RAD51 (all following polymorphisms summarized in Table 2) [53]. More recently, Ozmen et al. investigated the sequence variations of BRC1-BRC8 and C-terminus of canine BRCA2 [54] and found multiple SNPs in exon 11 and one insertion/deletion polymorphism in exon 27. Further in silico investigations added to speculation that a specific variation in BRC3 is the most likely to affect the RAD51 binding strength. Consequently, variations affecting the binding of RAD51 are critical, as they do not allow a proper BRCA2-RAD51 binding and further provoke an impaired repair through homologous recombination [54].

**Table 2:** Variations affecting the interaction with RAD51

Type of variation	Name/designation	Localization	Effect	Studied material	Reference
1 polymorphism	1425P allele	BRC3–4	Reduced binding strength with RAD51	Blood samples of tumor-free dogs	[53]
19 SNPs	amongst others, c.2383A > C (T1425P)	BRC1-BRC8, C-terminus region (exon 11)	Affects RAD51 binding strength	Tissue samples from canine mammary tumors	[54]
4 substitutions	S1078 W, A1108G, T1425P, T1559P	BRC3	Affects RAD51 binding strength	In silico analysis derived from mammary tumors	[54]

### Potential of applying this knowledge to canine mammary tumor therapy

Surgery is widely the most accepted treatment for dogs with mammary tumors, as it is the most effective for local tumor control [9, 55]. However, as a surgical treatment can be unsuitable because of the presence of metastases, chemo- and radiotherapy are reasonable additional therapies. There is, however, limited information about their efficacies in canine mammary tumors and tumors often present resistances [9, 12]. As a consequence, there is an interest to investigate additional treatments to increase the efficacy of chemo- and radiotherapeutics, and take advantage of mutations observed in mammary cancer in dogs. Therefore, the group of pharmacological drugs of PARP inhibitors could be investigated for treatment of mammary tumors in canine patients.

PARPi are inhibitors of the PARP1 protein, critical for SSB repair. If PARP1 is inhibited, SSBs induced by radiation or alkylating agents will be converted to DSBs during replication, eventually triggering cell death [24, 33, 56]. PARPi, e.g., olaparib, rucaparib, and niraparib, are compounds that “trap” PARP1 on DNA, preventing autoPARylation and PARP1 release from the site of damage. Consequently, they hamper the catalytic cycle of PARP1, and differ in their ability to trap, some being more potent than other PARPi [57–59]. PARPi were first used in human clinical trials testing the rucaparib/temozolomide combination in melanoma human patients [60] (following studies are summarized in Table 3). New studies arose based on preclinical data showing the synthetic lethality interaction between BRCA2 mutations and PARP inhibition [33, 56]. In 2009, a phase I clinical trial of olaparib was started, including ovarian and breast tumor patients (among other tumor types included) with germline BRCA1 or BRCA2 mutations. Sixty-three percent of the patients with mutations experienced a clinical benefit, thus showing the clinical effect of synthetic lethality using PARPi [61]. This was further confirmed with phase II trials [62–64] and as a consequence, olaparib was recently approved for ovarian cancer [65], followed by other PARPi such as rucaparib, niraparib and talazoparib [66–68]. Unfortunately, as with other targeted therapies, acquired resistance to PARPi therapy is observed in most patients with advanced cancer [24]. Mechanisms developed by cancer cells to resist include inactivation of DNA repair proteins [69, 70] and secondary mutations, both leading to the restoration of the homologous recombination function [71, 72] and in some cases, leading even to restoration of PARP1 [73].

**Table 3:** Summary of mentioned clinical studies involving PARP inhibitors

PARP inhibitor tested	Cancer type	Number of patients receiving the drug	Dose	Efficacy	Major side effects attributable to the drug	Reference
Rucaparib (/temozolomide)	Metastatic melanoma	46	150–200 mg/m2/day	Clinical benefit for 34.8% of the patients	Anemia (87%), constipation (48%), fatigue (54%)	[60]
Olaparib	Solid tumors (ovarian: 35%)	60	10 to 600 mg twice daily	Clinical benefit for 63% (in the BRCA mutations carriers patients)	Nausea (32%), fatigue (30%), vomiting (20%)	[61]
Olaparib	Breast	*Cohort 1*: 27	400 mg twice daily	ORR*: 41%	Fatigue (56%), nausea (56%), vomiting (22%)	[62]
		*Cohort 2*: 27	100 mg twice daily	ORR*: 22%	Nausea (41%), fatigue (30%),	
Olaparib	Ovarian	*Cohort 1*: 33	400 mg twice daily	ORR*: 33%	Nausea (48%), fatigue (33%), anemia (18%)	[63]
		*Cohort 2*: 24	100 mg twice daily	ORR*: 13%	Nausea (37%), fatigue (38%),	
Olaparib	Ovarian, breast, pancreatic and prostate	298	400 mg twice daily	Tumor response rate*: 26.2%	Fatigue (60%), nausea (60%), vomiting (37%)	[64]
Olaparib	Ovarian	223	400 mg twice daily	ORR*: 34%	Anemia (34%), nausea (64%), fatigue (66%)	[65]

ORR* (Objective Response Rate): according to RECIST, with confirmation of response at least 28 days apart by CT scan and RECIST. Tumor response rate*: according to RECIST, with confirmation of response at l east 28 days apart

Another combination therapy has been shown to interfere with DNA repair pathways: hyperthermia. Controlled heat applied to tumors prior to radiotherapy is used clinically to increase the efficiency of the radiation treatment. Amongst other changes in the microenvironment including increased blood perfusion [74–76], one reported cellular effect of hyperthermia is the inhibition of DNA repair mechanisms. There is evidence of the influence of heat on several DNA repair pathways, including the homologous recombination pathway [77–79]. Krawczyk et al. demonstrated that mild hyperthermia (41 °C applied with an incubator for a duration of 60 min) inhibits homologous recombination: they showed in particular that hyperthermia delays formation of IRIF (irradiation induced foci) by RAD51 and BRCA2 proteins, possibly by inducing temporary but robust degradation of BRCA2 [79]. Therefore, degradation of BRCA2 by heat carries the promise that PARPi could be successfully used in much broader patient populations, as it will temporarily inactivate homologous recombination, regardless of the patient’s genetic background [78, 80, 81]. Oei et al. recently investigated triple modality therapy using hyperthermia, radiotherapy and PARPi in BRCA2-proficient and -deficient mouse cell lines. In all cell lines tested, the addition of heat to radiotherapy and PARPi resulted in the lowest cell survival, the highest levels of DNA damage and apoptotic levels compared to duo-modality treatments [81].

As of today, no PARPi has been approved or is routinely used for the treatment of cancer in dogs. HowSaba et al. reported in 2016 their investigations on canine treatment with PARPi in a study using iniparib [82] (we must mention, though, that iniparib was reportedly shown not to be a bona fide PARPi [83–85]). In their work, inaparib could be safely administrated to dogs: they were treated with inaparib alone and in combination with carboplatin. Plasma and tumor tissue samples were collected before and at several times after treatment in order to perform pharmacokinetic (PK) and biologic analysis.

Additionally, although PARPi have extended patients’ progression-free survival in the clinical trial setting, they are also associated with high costs. For instance, in 2017, a 30-day supply of olaparib amounted to $13,000, plus additional costs for therapy monitoring and management of adverse events [86]. To investigate the benefit of adding PARPi therapies that are efficient - to a certain point, as resistances are common and develop through multiple mechanisms - but also costly and toxic, researchers have performed studies on their cost-effectiveness [86, 87]. In 2018, Zhong et al. showed that olaparib and niraparib may not be cost-effective treatments; indeed, they determined an ICER value (Incremental Cost-Effectiveness Ratio, a statistic tool which summarizes the additional cost of an outcome gained by one intervention compared with another) of $250,000 per PFS (Progression-free survival) life-year [86]. When considering a reference value of society’s willingness to pay of $100,000 per PFS life-year, olaparib and niraparib are not considerable options.

Toxicities of the different available PARPi overlap, and some differences exist. The most common adverse side effects include anemia, fatigue, nausea and neutropenia [88]. For instance, the SOLO2/ENGOT-Ov21 phase 3 trial testing olaparib resulted in patients suffering from anemia (18%), fatigue (4%), neutropenia (4%) and abdominal pain (5%). To limit the seriousness of the adverse effects, the researchers interrupted doses (45%), reduced them (25%) or discontinuated treatment (11%) [89]. PARPi are not completely harmless and show a diversity of relevant adverse effects. However, as previously mentioned, Saba et al. showed that iniparib could be administrated safely to dogs, at above dosages comparable to those used in humans [82]. In the 19 dogs, toxicity did not increase beyond carboplatin toxicity alone. However, more clinical studies administrating PARPi to treat breast or ovarian canine cancer are clearly needed to rightly report PARPi-related adverse effects in canine patients.

Finally, PARPi would represent an additional cost to the treatment of dogs against cancer, which is a factor should not be overlooked. For a dog, the treatment would cost the owners an additional $10,000–15,000 per month. Unfortunately, the majority of pet owners do not have insurance for their dog and have to bear the costs on their own. As chemo- and radiotherapy for canine patients often amounts to several thousand dollars, this already represents a burden for the owners of these patients. Therefore, the value of using PARPi for canine patients is questionable due to financial reasons, especially without a larger efficiency added to the common therapy.

## Conclusions

There is evidence that canine BRCA2 gene alterations are associated with mammary tumors. Indeed, mutations in the BRCA2 gene were found in dogs, and they seem to affect interactions with RAD51 and impact DNA repair [22, 23, 35]. Reduced expression levels of BRCA2 have been evidenced in canine mammary tumors [90, 91] and are caused by different reported mechanisms. First, mutations of BRCA2 lead to different genetic variations of the gene and disturb mRNA levels [35, 36]. Second, non-sense mediated mRNA decay is described to be involved in the reduction of BRCA2 expression [23, 37]. Thus, polymorphisms in the canine BRCA2 gene, in particular in the NLS regions, are involved in the mislocalization of BRCA2. Furthermore, variations in NLS2 were shown to affect translocation of BRCA2 and were associated with the morbidity rate of the studied dogs [39]. Additionally, polymorphisms in the BRC repeats region (where the interaction with RAD51 takes place) of canine BRCA2 are reported [43]. These variations are critical: without a proper binding of BRCA2 and RAD51, repair through homologous recombination is impaired [54].

PARPi compounds, i.e. olaparib, rucaparib, and niraparib, are based on the synthetic lethality interaction between BRCA2 mutations in some patients, and PARP inhibition [24, 33, 56]. First investigations in dogs show that one type of PARPi, iniparib, is tolerable to them [82]. Unfortunately, no more investigations about use of PARPi in canine patients have been performed to date. Before these therapies are administered in combination with chemo- and radiotherapy in animal clinics, more investigations are needed. In addition, PARPi represent a non-negligible addition to the already-high cost of cancer treatment for owners of dogs.

Nevertheless, mutations of the BRCA2 gene in dogs can be exploited for both diagnosis and treatment of mammary tumors in canine patients and to further advance cancer treatment in veterinary oncology.

## Data Availability

Not applicable.

## Abbreviations

BRCA1/2: Breast Cancer genes 1 and 2
DSB: Double-Strand Break
ICER: Incremental Cost-Effectiveness Ratio
LOH: Loss of Heterozygosity
NLS: Nuclear Localization Signal
NMD: Nonsense-Mediated mRNA Decay
PARP: Poly(ADP-Ribose) Protein
PARPi: PARP inhibitor
PFS: Progression-Free Survival
PK: Pharmacokinetic
PTC: Premature Termination Codon
RPA: Replication Protein A
SNP: Single Nucleotide Polymorphism
SSB: Single-Strand Break
